# Medical History from the Earliest Times

**Published:** 1893-06-10

**Authors:** E. T. Withington


### MEDICAL HISTORY FROM THE EARLIEST TIMES.

By E. T. WITHINGTON, M.A., M.B. Oxon.

### LVII.—THE CLINICIANS.

IN the preceding chapters we have seen how able physicians, led astray by love of system, attempted to replace the ruined fabric of Galenism by new edifices based upon the still but half-solidified foundations of physics and chemistry. We shall now see how others fell back upon an older and a better model, and how the banner of the Hippocratic medicine, unfurled in England by Thomas Sydenham, was carried to Italy and Holland by his admiring disciples, George Baglivi and Hermann Boerhaave.

The praises of Sydenham (1624-89) have been recorded by so many able writers, and his works are so readily accessible, that he may be here discussed more briefly than his merits would otherwise demand. The first and greatest of these merits was that he not only repeated the Hippocratic dictum that medicine depends not on theory but on observation, as Sylvius and others had done before him, but that he also carried it out in practice. Thus, to take the example which most impressed his contemporaries, he substituted for the old treatment of fevers by purgatives and sweating, with the view of getting rid of a supposed morbid humour, the antiphlogistic method of cooling drinks, fresh air, and venesection as being most in accordance with Nature and experience. The progress of medicine may, he thinks, be furthered in three ways: (1) By accurate descriptions or natural histories of diseases, written without any pre-conceived idea, separating them into their various species, and distinguishing the essential from the accidental symptoms; (2) by establishing a fixed principle or method of treatment, founded upon experience; (3) by searching for specific remedies, which he believes must exist in considerable numbers, though he admits that the only one yet discovered is Peruvian bark. Sydenham holds that diseases are as typical and distinct from each other as the various species of plants and animals, which they resemble in appearing and disappearing with the seasons; and though he believes with Hippocrates that they are efforts of Nature towards a cure by the elimination of morbific matter, he compares them at the same time to parasites which develop in man owing to some change in his humours, just as, to use his own simile, mosses, mushrooms, and mistletoe grow on trees the juices of which have been corrupted by some internal or external agency. The progress of medical knowledge has shown that he exaggerated both the number of specific remedies and the specific nature of disease, and this is still more the case with his favourite doctrine of the "epidemic constitution," which he borrowed directly from Hippocrates. According to Sydenham, acute diseases, especially those of an epidemic character, while differing from one another in special properties, present a general type which varies from time to time, not through change of climate or season, but "because of certain secret and inexplicable alterations in the bowels of the earth," and a knowledge of the existing type is of fundamental importance in treatment. This theory, though perhaps not entirely false, did great harm, for it became the fashion to ascribe excessive mortality of any kind to a "genius epidemicus," "cosmo-telluric miasm," or mysterious influence, sometimes confined to a single hospital ward, at other times spreading itself over whole countries, a doctrine which not only saved the physician the trouble of further investigation, but also excused his want of success, for what can mere mortals do against cosmo-telluric influences and epidemic genii? But we need not dwell on the unavoidable errors of a great man, and the influence of Sydenham's teaching as a whole was as beneficial as it was extensive.

Chief among his pupils in spirit, if not in fact, were Baglivi (1668-1707) and Boerhaave (1668-1738). The former, who flashes like a bright meteor across the horizon of medical history, has been called an Italian Sydenham, and had he lived, would doubtless have merited the title; but he may be better compared with Bichat, whom he closely resembled in his wonderful activity and early death, as well as in the style and nature of his teaching. Having studied at Naples, and graduated perhaps at the ancient University of Salerno, Baglivi went round the schools and hospitals of Italy to observe the state of medical teaching and practice, lingering especially at Bologna, where he attended the lectures of Malpighi, then the greatest of living anatomists. He published the results of his observations and experience in his "Praxis Medica," and was soon afterwards elected Professor of Anatomy at Rome, and, what he considered a still higher honour, foreign member of the Royal Society, in succession to Malpighi, 1697. His few remaining years were spent in the continuous labours of teaching, research, and practice. He lectured twice daily in anatomy, besides giving instruction in chemistry and medicine. He devoted much time to experimental physiology, which he declared was the source of all the chief discoveries of the age, and he never went to bed till he had written an exact account of the condition of each of his numerous patients. This excessive activity gave rise to several illnesses, and probably hastened his death (June 17th, 1707), at the early age of 38.

Baglivi has left us two important works, the "Praxis Medica" and the "De Fibra Motrice," devoted respectively to the art and science of medicine. The latter is of much value, and gave origin to the so-called "solidar" pathology, but space only permits some brief extracts from the former, one of the most interesting books of the 17th century. Here are a few of the aphorisms in which it abounds: "The physician is the servant and interpreter of Nature." "Medicine consists entirely in observations." "The healing art is not a product of human speculation, but is the daughter of Time." "If anywhere, certainly in medicine, is it required to know much and to do little, especially in acute and complicated cases, and we should try to remove the prejudice of many patients who think their cure depends on the amount and variety of the drugs given them." "The old proverb, 'Order the bricks to the line, and not the line to the bricks,' applies exactly to physicians, who must adapt their theories to that immutable plumb-line suspended in the universe by the hand of God, even the laws of Nature, which will not deviate a hair's breadth to suit our fallacies.'

One of the chief hindrances to progress in medicine is, says Baglivi, the love of system making. Both "chemists" and "mechanics" have put forward ingenious theories as to the nature of fever, but without the slightest practical effect upon its treatment, which has in the meanwhile been almost revolutionised by the careful clinical observations of "Thomas Sydenhamius artis nostrae ornator et ornamentum." Though Baglivi holds with Hippocrates that prognosis is higher than diagnosis, since it is better to know what is likely to become of a patient than to be able to give a correct name to his disorder, he still maintains that the latter art is too much neglected, and he especially attacks the rough and ready division of fevers into simple and malignant. As some aid towards a better classification he describes what he calls mesenteric fever, an acute epidemic disease common at Rome, and characterised by inflammation of the intestine and mesenteric glands. It runs a prolonged course, rarely presents any definite crisis, is in no way benefited by bark, and demands a patient, temporising, and expectant treatment. The medical reader will have no difficulty in recognising the first clear description of what is now called typhoid or enteric fever. The following is Baglivi's estimate of the medical value of three important beverages then recently introduced. "Excessive coffee drinking causes headache, sleeplessness, and tremor. I saw two patients so affected who immediately recovered on leaving off drinking it. Speaking of coffee I may notice in passing that it is a sovereign cure for after-dinner headache. I have observed this in numerous patients at Rome, and also in my own case; for through excessive study and the labours of practice I got an indigestion, with headache and heaviness, about three hours after dinner, so] I took three cups of coffee, which acted like a charm. I also tried tea and chocolate, but with less effect. Tea, however, is useful in headaches from other causes, especially nervous; while chocolate tends to increase the quantity of blood, and is a nutritious tonic for the debilitated. But its excessive use may cause plethora and a liability to inflammatory disorders and apoplexy, whence, perhaps, the frequency of those diseases in our days." Baglivi applies the Baconian apologue of the ant, the spider, and the bee to the various medical sects. "The ant, who gathers food at random and uses it immediately, is a type of the empiric who goes hither and thither collecting facts, and applies them at once, untested either by the touchstone of experience or the crucible of reason. The spider, who spins her whole web out of herself, represents the theorising doctors, the pure dialecticians of science. The bee does better than either, for she gathers the crude honey from the flowers, introduces it into her own organism, and there brings it to all the perfection of which it is capable. But if you look for physicians who act like her you will find none." The medical historian, however, may fairly point to Baglivi himself as an example of this best type of physicians, for he has not only left us the nutritious honey of his teaching, both practical and theoretic, but he improved the few bright hours allotted to him with a diligence worthy of our highest admiration.

